# Splenic Transcriptional Responses in Severe Visceral Leishmaniasis: Impaired Leukocyte Chemotaxis and Cell Cycle Arrest

**DOI:** 10.3389/fimmu.2021.716314

**Published:** 2021-11-05

**Authors:** Caroline Vilas Boas de Melo, Felipe Guimarães Torres, Micely D’El-Rei Hermida, Jonathan L. M. Fontes, Bianca Ramos Mesquita, Reginaldo Brito, Pablo Ivan P. Ramos, Gabriel R. Fernandes, Luiz Antônio Rodrigues Freitas, Ricardo Khouri, Carlos Henrique Nery Costa, Washington L. C. dos-Santos

**Affiliations:** ^1^ Laboratório de Patologia Estrutural e Molecular (LAPEM), Instituto Gonçalo Moniz, Fundação Oswaldo Cruz, Salvador, Brazil; ^2^ Laboratório de Enfermidades Infecciosas Transmitidas por Vetores (LEITV), Instituto Gonçalo Moniz, Fundação Oswaldo Cruz, Salvador, Brazil; ^3^ Centro de Integração de Dados e Conhecimentos para Saúde (CIDACS), Instituto Gonçalo Moniz, Fundação Oswaldo Cruz, Salvador, Brazil; ^4^ Informática de Biossistemas, Instituto René Rachou, Fundação Oswaldo Cruz, Belo Horizonte, Brazil; ^5^ Departamento de Medicina Comunitária, Instituto de Doenças Tropicais Natan Portella, Universidade Federal do Piauí, Teresina, Brazil

**Keywords:** visceral leishmanaisis, white pulp remodeling, spleen disorganization, transcriptomic (RNA-Seq), spleen pathology, hamster

## Abstract

Structural changes in the spleen have been reported in several infectious diseases. In visceral leishmaniasis (VL), a severe parasitic disease caused by *Leishmania* spp., the loss of white pulp accompanies a severe clinical presentation. Hamster model reproduces aspects of human VL progression. In the early stages, a transcriptomic signature of leukocyte recruitment was associated with white pulp hyperplasia. Subsequently, impaired leukocyte chemotaxis with loss of T lymphocytes in the periarteriolar lymphoid sheath occurred. This differential gene expression was subsequently corroborated by transcriptomic profiling of spleens in severe human VL. At the latest stage, spleen disorganization was associated with increasing clinical signs of VL. White pulp disruption was accompanied by decreased *DLK1* expression. The expression of *CXCL13, CCR5, CCL19, CCR6, CCR7* and *LTA* decreased, likely regulated by *CDKN2A* overexpression. Our findings enlighten a pathway implying cell cycle arrest and decreased gene expression involved in spleen organization.

## Introduction

The spleen plays a central role in the pathogenesis of visceral leishmaniasis (VL), a severe parasitic disease caused by *Leishmania infantum* or *Leishmania donovani* (1–4). In VL, infection in the spleen is persistent. This organ presents important sequential morphological changes that parallel the general clinical state of disease progression (5–7). The lymphoid follicles located in splenic white pulp (WP) respond to *Leishmania* infection by forming the germinal center and increasing B cell differentiation. This hyperplasia in the WP is accompanied by increasing numbers of *Leishmania*-infected macrophages, which leads to splenomegaly and increased parasite burden in later stages of disease (8). The enlargement of the spleen exacerbates its function of blood cell recycling, contributing to pancytopenia (9, 10). As the disease progresses, cell populations become substantially altered in the white and red pulp (RP). Lymphoid follicles decrease in size and the boundaries between the germinal center mantle area and marginal zone become less evident, eventually disappearing (6, 11–13).

Disruption in splenic microarchitecture has been reported in a variety of diseases, such as leishmaniasis and malaria, as well as many viral diseases, including HIV and COVID-19 (14–17). However, the mechanisms underlying these changes have yet to be elucidated. The splenic architecture is chemically maintained by a complex network of transcripts. In *Leishmania*-infected dogs with disorganized spleens, decreased *CXCL13* expression has been associated with a loss of follicular dendritic cells and decreased numbers of B cells in lymphoid follicles (18). Concomitantly, the accumulation of plasma cells in the RP may result from interference in B cell differentiation pathways in the spleen (13). Moreover, the overexpression of *CXCL12*, *BAFF* and *APRIL* in splenic RP supports the hypothesis of anomalous homing and increased plasma cell life span (13). In the periarteriolar lymphoid sheath (PALS), T cell apoptosis driven by cellular exhaustion markers, such as CTLA-4, may lead to decreased numbers of CD4+ T lymphocytes, which has been associated with WP disorganization (18–20).

Hamsters are known to develop a progressive form of VL that can be fatal, recapitulating many aspects of the disease observed in susceptible dogs and humans (21, 22). In addition to being associated with a mixed Th1 and Th2 response profile, susceptibility has also been linked to a failure to express the gene coding for nitric oxide synthase (*iNOS*), even in response to IFN-γ, which implies the inability of macrophages to control replication of intracellular parasites (23, 24). Although experimental infection in hamsters represents a suitable model for the study of lesions associated with severe forms of VL, the analysis of pathways involved in lesion development has been limited by a lack of compatible reagents. However, recent gene expression studies have substantially contributed to our understanding of mechanisms involved in immune response to VL (25, 26).

The present study demonstrates that *L. infantum* infection leads to disorganization of the spleen compartments in hamsters with severe clinical presentations of VL. We employ large-scale gene expression analysis to identify transcripts potentially involved in WP disruption. We propose that the histological changes seen during the course of infection correlated with the transcriptional differential gene expression and tissue distribution of T lymphocyte marker and DLK1 protein. We then corroborate our findings from experiments performed in hamsters using splenic transcriptomic profiles of severe human forms of VL.

## Materials And Methods

### Ethics Statement

All experimental procedures were approved by the Institutional Review Board of the Gonçalo Moniz Institute (IGM-FIOCRUZ, license nos. 004/2013 and 017/2015 for experiments performed in hamsters, and 3.491.092 for humans), and were carried out in accordance with Brazilian legislation on ethical animal experimentation. All patients were informed and consented to participate in the study.

### Hamsters and Infection Procedures

Golden Syrian hamsters (*Mesocricetus auratus*) were obtained from a FIOCRUZ animal care facility. Male hamsters, 6-8 weeks old, weighing 114 ± 20.2 g, were kept in cages under a controlled physiological light and temperature conditions. Each hamster was individually identified by a subcutaneous chip that was recognized by compatible reader (Microchip Partners). Injections were performed by intraperitoneal route in 1mL of either saline solution (control group) or a parasite suspension (infected group) containing 10^7^ promastigotes of *L. infantum* (strain MHOM/BR2000/Merivaldo2). *L. infantum v*irulence was maintained through passages in hamsters, and parasites were grown until reaching stationary phase *in vitro* in Schneider’s complete medium (Schneider + 20% fetal bovine serum [FBS], Gibco, USA) in a B.O.D. incubator at 24°C. Hamsters were euthanized by anesthetic overdose (10mg cetamin + 1mg xylazine/mL) at 30, 60, 120 and 150 days post-injection (dpi). Animal spleens were collected, weighed, and fragmented into three pieces for histopathological analysis, parasite culturing and RNA extraction. For parasite detection, macerated spleen fragment was sown in biphasic culture medium (blood agar + complete Schneider medium) in a B.O.D. incubator at 24°C and examined for up to 4 weeks.

### Clinical Signs, Serum Biochemistry and Hematology

Clinical signs of VL (presence of skin lesions, hair and weight loss, splenomegaly) were examined on a weekly basis. At the day of euthanasia, blood was collected by heart puncture. Blood was preserved in EDTA blood collection tubes and submitted to analysis of total red blood cell (RBC) counts and total and differential white blood cell (WBC) counts using an automated cell counter. For biochemical analysis, the blood was collected in serum collection tubes and centrifuged at 2,000 x*g* for 15 minutes at room temperature and serum was submitted to biochemical analysis of total serum protein, albumin and globulin fractions.

### Human Spleens

Human spleen samples were obtained from the Natan Portella Tropical Disease Institute (Teresina, Piauí, Brazil) following therapeutic splenectomy in patients with severe VL, two of whom were also coinfected with HIV. Both coinfected VL-HIV patients had undetectable viral load. Splenic material from two males and one female, median age of 51 [range: 45 – 55] years, were included in the study. Spleen fragments were submitted to histopathological analysis and RNA extraction.

### Histopathology and Morphometry

Spleen was collected during necropsy and fixed in an alcoholic acid formalin solution for 24-48h at room temperature. Tissues were paraffin-embedded prior to 3-4 mm sectioning and stained in hematoxylin & eosin. All tissue specimens were examined by two pathologists (WLCdS and LARF) on a group-blinded basis to estimate the intensity of inflammatory infiltrate, fibrosis and cell degeneration, as previously described (27). Architectural organization was assessed in the spleen samples according to Hermida et al. (3). In brief, well-organized or type 1 spleen was considered when WP microenvironments were easily distinguished in lymphoid follicles, the germinal center, PALS, mantle zone and marginal zone; slightly disorganized or type 2, corresponded to the loss of some boundaries between the WP microenvironments due atrophic or hyperplastic alterations; extensively disorganized, or type 3, was considered when boundaries were poorly delimited between WP and RP regions, and lymphoid follicle atrophy was observed (3, 6). Morphometric evaluations were performed to estimate WP/RP ratios using ImagePro Plus (Media Cybernetics).

### Immunohistochemistry and Morphometry

Spleen fragments were fixed in 10% formalin and embedded in paraffin; 4 μm-thick sections were obtained and mounted on silanized slides. The slides were then deparaffinized and rehydrated. For CD3 staining, the slides were immersed in a 10% ammonium hydroxide solution for 10 minutes to remove formaldehyde pigment, 3% hydrogen peroxide for 10 minutes to block endogenous peroxidase and in Tris-EDTA pH 9.0 for 30 minutes at 96°C to perform antigenic recovery. Slides were then cooled to room temperature for 20 minutes, and nonspecific staining was subsequently blocked by 5% bovine serum albumin for 20 minutes. For DLK1 staining, the slides were incubated with 20% ammonium hydroxide in 95% alcohol for 20 minutes, washed in running tap water, followed by washing in distilled water. Antigen recovery was performed by heating (115°C) in citrate buffer (pH 6.0) for 20 minutes. Endogenous peroxidase was blocked with 3% hydrogen peroxide in 3 incubations of 10 minutes, followed by washing with distilled water. Unspecific staining blocking was performed with horse serum (species of the secondary antibody) (Vector, 30022) for 15 minutes and washed in PBS, followed by permeabilization in PBS with 0.1% saponin for 5 minutes in 2 incubations. Afterwards, samples were incubated overnight with either anti-CD3 (1:300, ab16669, Abcam), rabbit anti-DLK1 (1:1000, ab21682, Abcam) or anti-CD20 (1:200, ab64088) primary antibodies, followed by incubation with the secondary antibody conjugated HRP (ab205718, Abcam) for 30 minutes for CD3 and CD20 staining, or anti-rabbit IgG polymer (Vector, 30026) for DLK1 staining. Staining was developed using a 0.02% 3,3-diaminobenzidine solution for 5 minutes, followed by nuclei counterstaining in Harris hematoxylin (Sigma). Spleen sections for CD3 staining were scanned at 200x magnification using an Olympus VS110 virtual slide scanning system (Olympus America Inc, United States). CD3 measurements were estimated in the five largest WP regions in seven cases per group, without overlapping areas. The boundaries of WP and PALS were established according to previously described morphological criteria (28, 29), and confirmed by staining for CD20 to visualize lymphoid follicles in WP. Images of the three hotspot areas of DLK1 staining were obtained at 400x magnification using Olympus BX53 camera in Image-Pro Plus software (V. 4.5, Media Cybernetics) and cell density was estimated. The same spleen specimens submitted to transcriptome analysis were used for DLK1 immunohistochemistry. Unfortunately, the specimens of 30 dpi timepoint, had a strong background that impaired the morphometric estimation. All measurements were morphometrically estimated using ImageJ software version 1.52 (National Institutes of Health, United States) following the delimitation of each respective region of interest. Additionally, the total area of CD3 staining was estimated in each compartment. DLK1 staining morphometry was estimated by two observers and the mean between the measures was represented. The means of cell density were used for statistical analysis.

### RNA Isolations

Spleen fragments collected from hamster dissection and biopsied humans were stored at -80°C and dissociated in Trizol (Invitrogen). Total RNA isolation was performed using the aqueous phase of Trizol mixture followed by purification using RNAeasy extraction kit (Qiagen, Inc.) according to the manufacturer’s protocol, including an extra step of DNA lysis through the addition of DNase. Total RNA purity was determined by NanoDrop spectrophotometer (ThermoFischer) and the concentration of the material was measured in Qubit fluorimeter (Invitrogen). Total RNA integrity was evaluated on an automatized electrophoresis Agilent Bioanalyzer 2100 (Agilent) with RNA 6000 Nano kit (Agilent). Samples were considered of high quality when RNA integrity number (RIN)>7.

### Hamster Spleen Transcriptomics

Large-scale expression data was obtained to identify the transcriptional response, including differentially expressed genes, between infected and non-infected animals in different time-points (30, 60, 120 and 150 days). Additionally, *L. infantum* RNA counts were also assessed. Once a total RNA was obtained that met the purity, concentration and integrity criteria, a contaminant depletion step (globins and ribosomal RNAs) was applied and the resulting sample was converted into cDNA and subjected to NextSeq platform (Illumina, Inc), according to the manufacturer’s protocol. An average of 30,000,000 readings per sample was expected. The sequencing was performed using paired-end strategy with fragment library of size 2×75 bases using stranded protocol. Transcriptomic profiles from the experimental VL samples were obtained using three biological replicas for each group (control and infected) at each of the four timepoints studied. The sample selection for transcriptomic analysis was based on the disruption of the splenic WP throughout the follow-up of the infection. The quality of reads was assessed by FastQC (v0.10.1) in each sample, followed by summarization of the reports using MultiQC (30). Golden hamster (Genome ID 11998) or *Leishmania infantum* (Genome ID 249) reference genomes were obtained from the NCBI Genome database and then aligned using STAR (31). The number of reads per transcript were calculated by FeatureCounts. Gene annotation was performed and differential expression (DE) was determined using data from the GEO repository (NCBI). Differential expression analysis was conducted using edgeR (32). Wald’s test was performed using DESeq2 (25). Functional analysis was carried out using Ingenuity Pathway Analysis (IPA) software (Qiagen, Inc.) considering a cut-off log fold change (FC) value of 2, false discovery rate (FDR) <0.05 and absolute z score of ≥2 (26). Unsupervised analysis was employed at each timepoint for the evaluation of canonical pathways and enrichment of diseases and functions (**Tables S1**, **S2**). Enrichment analysis of differentially expressed genes was performed overlapping networks for each timepoint. To investigate transcripts associated with spleen morphology and WP organization, a supervised analysis was performed with the addition of a panel of transcripts, followed by an unsupervised network expansion to evaluate interaction pathways related to splenic organization.

### Human Spleen Transcriptomics

Gene expression was assayed using nCounter platform (NanoString Technologies, Seattle, Washington), based on direct molecular bar codes coupled to target RNA transcripts using digital detection at the Genomics Core Leuven (VIB/KULeuven - Belgium). Two probes were employed, one that captures the mRNA of interest for the complementary sequence, while the other is connected to a fluorescent barcode that identifies the target by specific hybridization. The probes are mixed with the genetic material and transcripts of interest are identified by fluorescent barcodes. Samples from healthy spleen tissue were reanalyzed and obtained from the GTEx project (33), which performed whole-transcriptome profiling of multiple human tissues, including spleen. Three samples were included for this analysis, and raw reads from whole-transcriptome profiling were obtained from the SRA selector at http://trace.ncbi.nlm.nih.gov/Traces/study/?acc=phs000424. These samples were chosen based on their age and gender profile similarity with our infected tissue human donors. Gene annotation was performed using the biomaRt library for R (34). Mapping of the raw reads against the human genome reference (GRCh38) was performed using STAR (31) at default parameters, and HT-seq count was used to assign and count mapped reads to annotated genomic features (35) using GENCODE v. 25 annotations. Nanostring expression was quantified by counting the amount of mRNA identified in a single reaction. The present study constructed a panel consisting of 9 genes for human analysis (**Table S3**). Raw data were preprocessed using nSolver 2.0 software (NanoString Technologies).

### Statistical Analysis

The statistical analysis of transcriptomic data was performed as described above. Absolute numbers, means, medians and percentages are indicated in the text, tables and graphs. Comparisons between control and infected groups were performed using the t test or Mann-Whitney test as indicated. To perform comparisons between control and infected groups at each time point, ANOVA was used in combination with multiple comparisons by the Kruskal-Wallis test. Statistical significance was considered when p<0.05.

## Results

### Clinical Evaluation

The general characteristics of the animals are presented in **Table 1**. All infected animals presented positive spleen culture throughout all time points, and RNA copies aligned to *Leishmania infantum* presented an increasing trend over time of infection. Clinical signs of disease were present after 120 dpi in two animals (one had a crust on the snout, and another presented an ulcer in the oral region) and more intense in two animals at 150 dpi (one had cachexia, irregular breathing, dehydration, an ulcer in the oral region and pleural effusion; another was emaciated and presented an ulcer in the oral region). Statistically significant differences in clinical presentation were observed between the groups at 150 dpi (**Table 1**).

**Table 1:** Evidence of infection, clinical and laboratorial data from hamsters experimentally infected with *L. infantum*.

N	DAYS POST-INFECTION							
	30		60		120		150	
	CT	INF	CT	INF	CT	INF	CT	INF
	7	7	7	7	7	7	7	7
***Evidence of infection:***								
Spleen culture	0/7 (0%)	7/7 (100%)	0/7 (0%)	7/7 (100%)	0/7 (0%)	7/7 (100%)	0/7 (0%)	7/7 (100%)
Parasite RNA counts	76 [73–80]	53 [49–5180]	83 [50–112]	4162 [954–37669]	99 [75–145]	1046 [483–4845]	110 [99–119]	11509 [4766–403131]
***Clinical signs of disease:***								
Present	0/7 (0%)	0/7 (0%)	0/7 (0%)	0/7 (0%)	0/7 (0%)	2/7 (29%)	0/7 (0%)	2/7 (29%)
Number of signs*	0 [0–0]	0 [0–0]	0 [0–0]	0 [0–0]	0 [0–0]	0 [0–1]	0 [0–0]	4 [2–4]^a^
Body weight (Δ)	8.6 ± 2.2	0.3 ± 6^b^	29.9 ± 11.1	20.9 ± 5.3	41.2 ± 12.1	25.9 ± 12.7	38.8 ± 12.4	0.7 ± 22.2^b^
***Laboratory parameters:***								
RBC	8.2×10^6^ ± 3.5×10^5^	8.4×10^6^ ± 4.5×10^5^	8.3×10^6^ ± 3.6×10^5^	8.3×10^6^ ± 1.9×10^5^	8.1×10^6^ ± 5.6×10^5^	8.6×10^6^ ± 6×10^5^	7.8×10^6^ ± 5.2×10^5^	6.9×10^6^ ± 9.5×10^5^
Hemoglobin	14.7 ± 0.6	15.5 ± 0.4	15.3 ± 0.5	15.4 ± 0.6	13.9 ± 1.8	14.8 ± 1.3	13.6 ± 0.7	11.7 ± 1.7
Hematocrit	44.2 ± 2	45.8 ± 1.6	45 ± 2.1	44.3 ± 1.3	44.6 ± 3.4	44.8 ± 3.4	41.5 ± 2.6	35 ± 5.5
MCV	54.2 ± 0.4	54.8 ± 1.4	53.8 ± 0.8	53.8 ± 0.7	55 ± 1.8	54.2 ± 0.4	53.5 ± 2.4	51 ± 1.7
Platelets	7.4×10^5^ ± 7.8×10^4^	6.7×10^5^ ± 6.2×10^4^	6.1×10^5^ ± 5.1×10^4^	6.8×10^5^ ± 6.3×10^4^	9.6×10^5^ ± 1×10^5^	5.5×10^5^ ± 2.1×10^5c^	3.8×10^5^ ± 6.7×10^4^	4×10^5^ ± 2.3×10^5^
WBC	2120 ± 739.6	1600 ± 367.4	3260 ± 427.8	2600 ± 784.9	3367 ± 1145	3457 ± 1261	4040 ± 945	1300 ± 519.6
Eosinophils	45.4 ± 49.8	60 ± 16.7	51 ± 15.6	45.3 ± 17.8	41.8 ± 13.4	41.8 ± 13.4	49.8 ± 26.2	13 ± 5.1
Neutrophils	972.2 ± 343.4	711.8 ± 156.6	1414 ± 251.4	1104 ± 359.3	1366 ± 474.1	1429 ± 564.3	1482 ± 383.3	475 ± 164.8
Lymphocytes	1069 ± 353.6	791.8 ± 214.7	1740 ± 231.5	1406 ± 432.8	1926 ± 649.4	1937 ± 668.2	2148 ± 567.8	799 ± 344.8
Monocytes	33 ± 11.8	33.2 ± 15.6	76.4 ± 26.4	44.8 ± 15	33.6 ± 11.4	50 ± 31.4	80.2 ± 35.2	13 ± 5.1
Total serum proteins	6.7 ± 0.5	6.8 ± 1	4.6 ± 1	4.5 ± 0.9	8.1 ± 0.6	8.4 ± 0.7	6.3 ± 0.9	6.8 ± 1.5
Albumin	3 ± 0.4	2.7 ± 0.4	1.9 ± 0.1	1.8 ± 0.2	4.1 ± 0.2	4.2 ± 0.3	3.4 ± 0.2	3.4 ± 0.6
Globulin	3.7 ± 0.3	4 ± 0.9	2.7 ± 0.8	2.6 ± 0.6	4 ± 0.5	4.2 ± 0.5	2.8 ± 0.8	3.3 ± 0.9
Albumin/globulin	0.8 ± 0.1	0.7 ± 0.1	0.7 ± 0.1	0.7 ± 0.09	1 ± 0.1	1 ± 0.1	1.2 ± 0.3	1 ± 0.2

Data presented as means ± standard deviation. CT, Control; INF, Infected; RBC, red blood cells; MCV, mean corpuscular volume; WBC, white blood cells. *Median and interquartile range of the number of clinical signs of VL (ulcers, crusting, cachexia, irregular breathing, dehydration, pleural effusion). **Δ**, difference between initial weight (prior to infection) and final weight (day of euthanasia). Statistical differences: ^a^control and infected group at 150 dpi, Kruskal-Wallis test (p,0.03). ^b^control and infected groups at 30 dpi (p,0.01) and 150 dpi (p,0.004), Mann Whitney test. ^c^control and infected group at 120 dpi, t test (p,0.001).

While weight was consistently lower in infected animals compared to controls, significant differences were only seen at 30 and 150 dpi. Despite an absence of statistical significance, differences were detected at 150 dpi in hematological parameters, with decreasing trends observed in average red blood cells (RBC) counts, hemoglobin, hematocrit, RBC mean corpuscular volume (MCV) and white blood cell (WBC) counts in the infected animals. Upon necropsy, splenomegaly was observed at 120 and 150 dpi (**Table 2**).

**Table 2:** Morphological changes in the spleens of hamsters experimentally infected with *L. infantum*.

N	DAYS POST-INFECTION							
	30		60		120		150	
	CT	INF	CT	INF	CT	INF	CT	INF
	7	7	6	7	7	7	7	7
Spleen weight (g)^a^	0.2 ± 0.05	0.1 ± 0.04	0.2 ± 0.07	0.2 ± 0.1	0.2 ± 0.05	0.5 ± 0.09^c^	0.2 ± 0.03	0.5 ± 0.2^c^
Perisplenitis	0/4 (0%)	0/7 (0%)	0/6 (0%)	1/7 (14%)	0/6 (0%)	0/7 (0%)	0/7 (0%)	4/7 (57%)^d^
***White pulp:***								
Relative size^a^	22 ± 4.7	22 ± 8.5	19 ± 4.9	17.4 ± 4.1	22 ± 8.5	11.4 ± 3.8^e^	12.7 ± 4.2	9.2 ± 4.4
***Disruption:***								
None	4/4 (100%)	7/7 (100%)	6/6 (100%)	7/7 (100%)	5/6 (86%)	2/7 (29%)	6/7 (86%)	2/7 (29%)
Present	0/7 (0%)	0/7 (0%)	0/7 (0%)	0/7 (0%)	0/6 (0%)	5/7 (71%)	1/7 (14%)	3/7 (43%)
Intense	0/7 (0%)	0/7 (0%)	0/7 (0%)	0/7 (0%)	1/6 (14%)	0/7 (0%)	0/7 (0%)	2/7 (29%)
***Lymphoid follicle:***								
Normal	4/4 (100%)	4/7 (57%)	1/6 (17%)	4/7 (57%)	3/6 (50%)	3/7 (43%)	4/7 (57%)	1/7 (14%)
Hyperplastic	0/4 (0%)	2/7 (29%)	2/6 (33%)	2/7 (29%)	1/6 (17%)	3/7 (43%)	0/7 (0%)	2/7 (29%)
Atrophic	0/4 (0%)	1/7 (14%)	3/6 (50%)	1/7 (14%)	2/6 (33%)	1/7 (14%)	3/7 (43%)	4/7 (57%)
Size score ^b^	0 [0 – 0]	0 [0 – 1]	-0,5 [-1 – 1,2]	0 [-0,2 – 1,2]	0 [-1 – 0.2]	0 [0 – 1]	0 [-1 – 0]	-1 [-1 – 1]
***Germinal center:***								
Normal	4/4 (100%)	5/7 (71%)	1/6 (17%)	2/6 (33%)	4/6 (67%)	5/7 (71%)	6/7 (86%)	2/7 (29%)
Hyperplastic	0/4 (0%)	2/7 (29%)	4/6 (67%)	4/6 (67%)	2/6 (33%)	2/7 (29%)	1/7 (14%)	1/7 (14%)
Atrophic	0/4 (0%)	0/7 (0%)	1/6 (17%)	0/6 (0%)	0/6 (0%)	0/7 (0%)	0/7 (0%)	3/7^f^ (43%)
Size score ^b^	0 [0 – 0]	0 [0 – 2]	1 [-0,2 – 1]	1 [0 – 1]	0 [0 – 1]	0 [0 – 1]	0 [0 – 0]	0 [-3 – 1]
***Granuloma:***								
Presence	0/4 (0%)	0/6 (0%)	0/6 (0%)	4/7 (57%)	0/6 (0%)	7/7 (100%)	0/7 (0%)	7/7(100%)
Intensity score ^b^	0 [0 – 0]	0 [0 – 0]	0 [0 – 0]	1 [0 – 2]	0 [0 – 0]	2 [2 – 3]^g^	0 [0 – 0]	3 [2 – 3]^g^

CT, Control; INF, Infected. Data presented as absolute and relative frequencies. ^a^mean ± standard deviation. ^b^median and 1^st^ and 3^rd^ quartiles. Statistical differences: ^c^control and infected hamsters at 120 and 150 dpi (p<0.0001), ANOVA test. ^d^control and infected groups at 150 dpi (p=0.003), ANOVA test. ^e^control and infected groups at 120 dpi (p=0.02), Kruskal-Wallis test. ^f^control and infected groups (p=0.006), Kruskal-Wallis test. ^g^control and infected groups at 120 dpi (p=0.0002) and 150 dpi (p=0.0009), Kruskal-Wallis test.

### Histological Evaluation of Spleen

**Figure 1:**
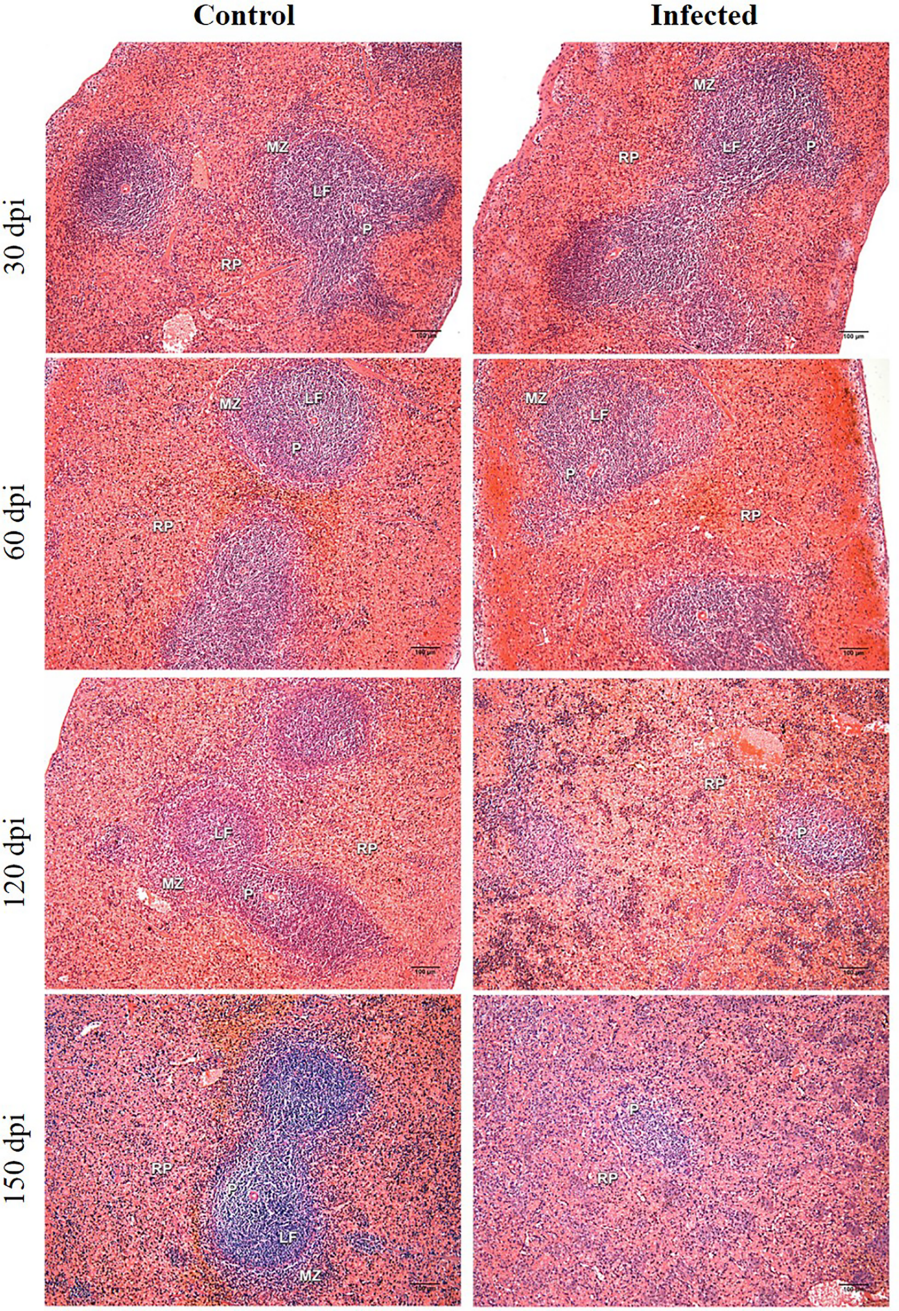
Histological analysis of hamster spleens. Representative photos of hamster spleens in control and infected groups (30-, 60-, 120- and 150 days post infection—dpi). H&E staining, 40x magnification, bar = 100 micrometers. Type I spleens are seen in all photomicrographs, except for infected at 120 dpi (type 2) and infected at 150 dpi (type 3). RP, red pulp; MZ, marginal zone; LF, lymphoid follicle; GC, germinal center; P,periarteriolar lymphoid sheath.

Chronic perisplenitis was observed in 4/7 infected animals at 150 dpi (**Table 2**). Smaller proportion of WP were seen in the *L. infantum*-infected animals compared to controls after 60 dpi, with statistically significant differences observed between the groups at 120 dpi. At 150 dpi, despite a persistent trend towards decreased WP size in the *L. infantum*-infected group, controls also presented less WP, possibly due to aging (**Table 2**). The disruption of WP architecture was observed in 10 *L. infantum*-infected hamsters, considered slight (Type 2) in 5/7 (71.4%) animals at 120 dpi and in 3/7 (42.8%) animals at 150 dpi, and intense (type 3) in 2/7 (28.6%) hamsters at 150 dpi (Mann-Whitney test, p=0.03) (**Table 2** and **Figure 1**). WP disruption was associated with germinal center atrophy in 3/7 (43%) infected hamsters at 150 dpi (**Table 2**).

Exclusively in infected hamsters, small granulomas were found at discrete (2/7, 29%) or moderate (2/7, 29%) intensity at 120 and 150 dpi in both WP and RP (**Table 2**).

### Transcriptomic Splenic Profiles

**Figure 2:**
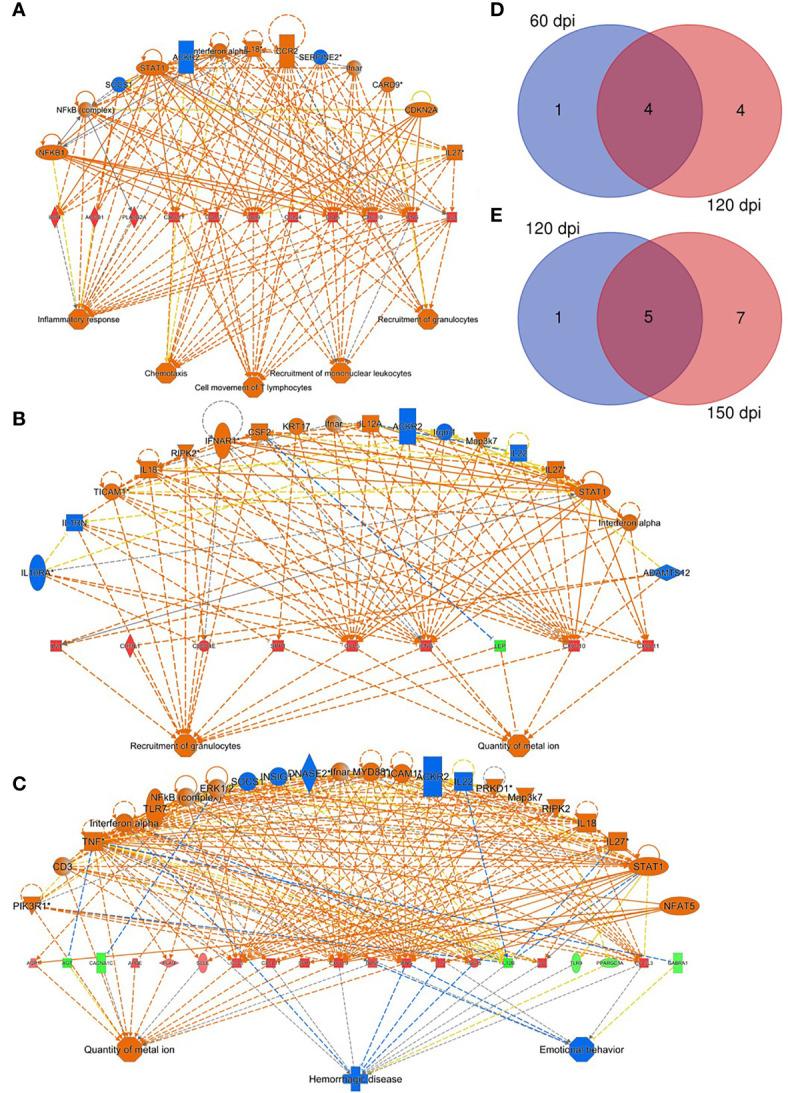
Regulatory effectors of splenic response to *Leishmania infantum* infection. **(A–C)** Network of transcripts associated with inflammatory response processes, chemotaxis, T lymphocyte movement, recruitment of mononuclear leukocytes and recruitment of granulocytes, quantity of metal ion, hemorrhagic disease and behavior in hamsters at 60 **(A)**, 120 **(B)** and 150 dpi **(C)**. Patterns of expression: green = low; red = high. Secondary interactions of transcripts and transcript-associated processes: orange= predicted activation; blue= predicted inhibition; dotted lines= indirect interaction; continuous lines= direct interaction; gray lines= no predicted effect; yellow lines= inconsistent findings. Venn diagrams depict number of overlapping transcripts expression at 60 and 120 dpi **(D)** and at 120 and 150 dpi **(E)**.

Based on the classification of WP organization, spleen tissues from uninfected (n=12) and infected (n=12) hamsters were submitted to RNA-seq analysis. Overall, the transcriptomic profile revealed an increase in differentially expressed (DE) transcripts during the course of infection (**Figure S1A**): 1040 (30 dpi); 1813 (60 dpi); 5874 (120 dpi) and 6512 (150 dpi). Principal component analysis pictures differential distributions in accordance with infection status and duration (**Figure S1B**). The distribution of groups along PC2 reflects some degree of splenic disorganization, while organized spleens are distributed along PC1 (**Figure S1B**). To investigate the functional effects of smaller groups of genes, an adjusted false discovery ratio (FDR) <0.05 and fold change (FC) of 2 were used to generate consistently expressed interaction networks at each time point. Under these parameters, only two transcripts were included in the analysis at 30 dpi, reflecting a lack of significant alterations in the histology of the spleen and the absence of clinical presentation at 30 dpi. However, at all remaining time points, higher numbers of transcripts were included making it feasible to build interaction networks. At 60 dpi, a set of overexpressed genes were predicted as regulator effectors for the functions of leukocyte trafficking and inflammatory process (**Figure 2A**). At this timepoint, genes related to granulocyte recruitment were also found to be overexpressed. At 120 dpi, the granulocyte recruitment remained activated, with a greater number of genes included (**Figure 2B**). At this time point the quantity of ion metal became altered, primarily stimulated by *SPP1*, *CCL5*, *IFNγ*, *LEP* and *CXCL10*. This effect was enriched to the last time point (150 dpi), revealing an increased number of associated transcripts (**Figure 2C**). Systemic alterations as in hemorrhagic disease and behavior were predicted in response to the set of transcripts expressed at this stage of the infection. Part of the differential expression of transcripts involved in the functional analysis was shared between time points 60 and 120 dpi (**Figure 2D**) and between 120 and 150 dpi (**Figure 2E**), reflecting a kinetics in the molecular signature.

### Transcriptomic Kinetics Processes

**Figure 3:**
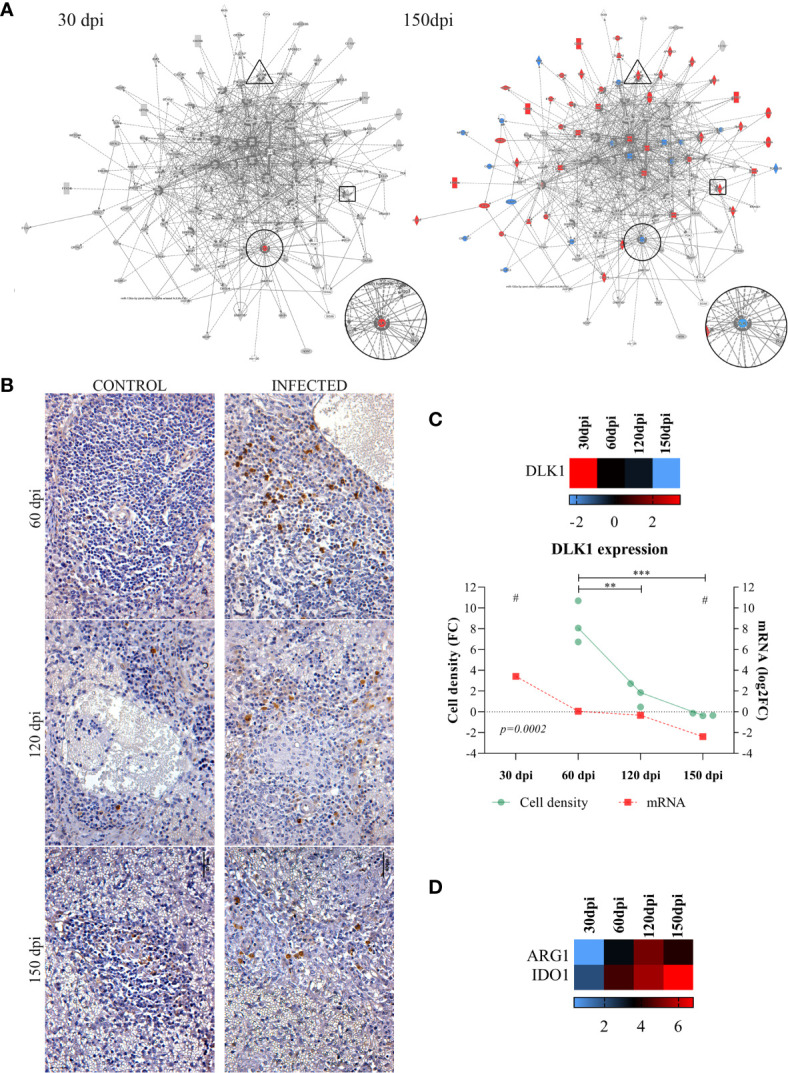
Common differentially expressed transcripts at different stages of *Leishmania infantum* infection. **(A)** Network of overlapping DE genes at 30 and 150 dpi. Pattern of expression in networks: blue = low; red = high; gray = no differential expression. *DLK1* is indicated in circles, *IDO1* in rectangles and *ARG1* in triangle forms. **(B)** Immunohistochemistry of DLK1 protein (brown staining) in the spleen of control and infected hamsters at 60, 120 and 150 dpi, 400x magnification. Bars= 40µm. **(C)** Scatter plots of morphometric estimates of DLK1 protein expression from immunohistochemistry staining in the spleens of infected hamsters at 60, 120 and 150 dpi (green line and dots). Cell density per area values were expressed in fold change (FC) from control hamsters. Expression of *DLK1* transcript evidenced by RNA-sequencing at 30, 60, 120 and 150 dpi (mRNA, red line and squares), and in heatmap represented by log2FC. Lines represent the mean of the values. **= statistical difference of cell density FC between 60 dpi and 120 (p=0.001) and ***= statistical difference of cell density FC between 150 dpi (p=0.0005), ANOVA, Tukey’s multiple comparison test. #= FDR for statistical significance of *DLK1* transcript expression at 30 and 150 dpi (padj<0.0001). Test for linear trend was performed to cell density FC between time points, with statistical significance for linear reduction at each timepoint (p=0.0002). **(D)** Expression of *ARG1* and *IDO1* at 30, 60, 120 and 150 dpi represented in log2FC in heatmap. Pattern of expression in heatmap: blue = low; red = high; black = no differential expression.

Given the importance of analyzing biological processes along the course of infection, we overlapped transcripts identified as differentially expressed onto enriched networks to elucidate key processes (**Figure 3**). At 30 dpi, as only two differentially expressed transcripts were identified after the functional analysis (FDR<0.05, FC of 2), only Delta Like Non-Canonical Notch Ligand 1 – *DLK1* was observed to be differentially expressed in common with the remaining timepoints. At 30 dpi, *DLK1* was observed to be upregulated (**Figure 3A**, 30 dpi). This expression pattern was reversed at 150 dpi (the latest stage of infection), when the downregulation of *DLK1* occurred (**Figure 3A**, 150 dpi). We performed an antibody-based validation of this transcript of DLK1 in the splenic tissue at 60, 120 and 150 dpi (**Figure 3B**). Cells expressing DLK1 were distributed around perivascular area. The density of DLK1-expressing cells decreased at each timepoint (p=0.0002), correlating with the decreasing expression of *DLK1* mRNA found in the transcriptomic analysis (**Figure 3C**) of the spleen of *Leishmania*-infected hamsters. With regard to gene expression analysis by overlapping along infection, transcripts deemed relevant to VL, such as *ARG1* and *IDO1*, exhibited high expression at 60, 120 and 150 dpi (**Figure 3D**).

### T lymphocytes and Spleen Disorganization

**Figure 4:**
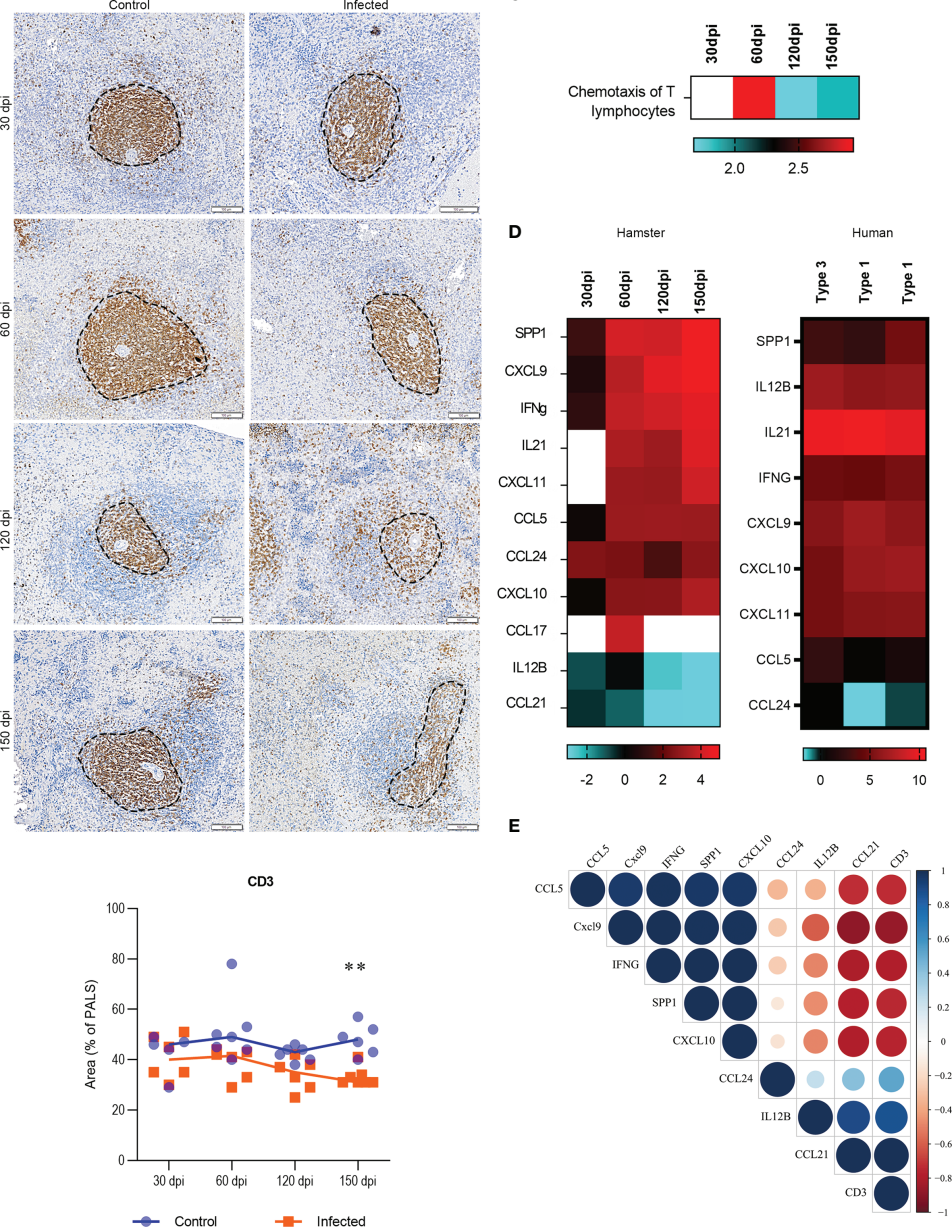
Transcriptomic and tissue profiling of T cells in spleens of *Leishmania infantum-*infected hamsters. **(A)** Immunohistochemistry of splenic CD3 (brown staining) in PALS area (dotted line) of control and infected hamsters at 30, 60, 120 and 150 dpi, 200x magnification. Bars= 100µm. **(B)** Scatter plots of morphometric estimates of CD3 expression in the spleens of control (blue) and infected (orange) hamsters at 30, 60, 120 and 150 dpi. Values were expressed in percentages of positive staining for CD3 within the PALS area. Lines represent the median of the values. **= statistical difference between control and infected groups at 150 dpi, p=0.004, Kruskal-Wallis test. **(C)** Heatmap representing predicted function of chemotaxis of T lymphocytes in the dataset (log2FC). **(D)** Set of transcripts that jointly predict the function of T lymphocyte chemotaxis in experimental (30, 60, 120 and 150 dpi) and human VL. Spleens from humans were organized (type 1) or disorganized (type 3). Patterns of expression: blue = low; red = high; blank = no detection. **(E)** Correlation matrix between transcripts that jointly predict the function of T lymphocyte chemotaxis (*SPP1*, *CXCL9*, *IFN-γ*, *CCL5*, *CCL24*, *CXCL10*, *CCL21* and *IL12B* – ) and CD3 expression in the spleen of infected hamsters at 30, 60, 120 and 150 dpi. Values were corrected by Log2 in comparison to respective control groups. Blue= positive correlations; Red= negative correlations. Color intensity and circle size are proportional to the correlation coefficient (*r*) of Pearson method.

Splenic T lymphocytes identified by CD3 staining were found on decreasing trend in area percentages of PALS in infected hamsters from 60 dpi (**Figures 4A, B**). Strikingly, when we performed an unsupervised enrichment of biological processes, chemotaxis of T lymphocytes emerged with the highest score in the dataset and consistently presented a pattern of differential expression over the course of infection (**Figure 4C**). The upregulation of the T lymphocyte chemotaxis pathway observed at 60 dpi decreased to the next timepoint (120 dpi), concomitant with decreases in PALS and WP areas. Impaired T lymphocyte chemotaxis persisted until 150 dpi and in association with statistically significant lower PALS area, indicating a potential transcriptomic mechanism linked to profound WP disorganization at later stages of disease. Genes associated with T lymphocyte chemotaxis included *SPP1*, *IL-21*, *CXCL9*, *IFN-γ*, *CXCL11*, *CCL5*, *CCL24* and *CXCL10*, which exhibited upregulation, as well as *CCL21* and *IL12B* which appeared downregulated (**Figure 4D**). Except for *CCL17* and *CCL21* that were not included in the nCounter target panel, all the transcripts involved in T lymphocyte chemotaxis were validated in the splenic transcriptome in human VL, of which included two organized (type 1) and one disorganized spleen (Type 3) (**Figure 4D**). Relevant correlations between tissue expression of CD3 and transcripts of the genes in the T lymphocyte chemotaxis pathway were observed, indicating a genetic regulation of splenic organization (**Figure 4E**).

### Cell Cycle Arrest and Spleen Disorganization

**Figure 5:**
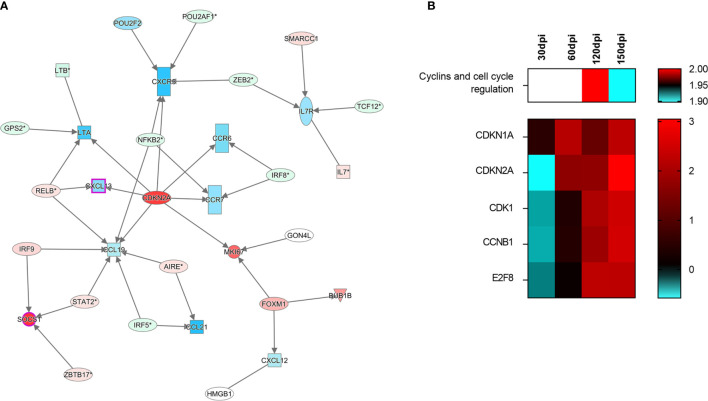
Signaling of cell-cycle arrest in the spleen of hamsters infected with *Leishmania infantum*. **(A)** Interaction network of transcripts associated with splenic white pulp disorganization at 150 days post-infection. Blue and red shading indicates differing grades of expression. *CDKN2A* can be found at the center of the network. **(B)** Canonical pathway of cyclins and cell cycle regulation at different stages of infection and set of transcripts involved in signaling. Patterns of expression: blue = low; red = high; blank = no differential expression.

In light of concurrent findings regarding impaired leukocyte recruitment and the histological disruption of spleen microenvironments, we then investigated expression patterns among the following set of genes known to participate in spleen compartment organization: *CXCL13*, *CXCR5*, *LTA*, *CCR7*, *CCR6*, *CCL19*, *CCL21*, *CXCL12* and *MKI67*. To evaluate transcripts associated with spleen morphology and WP organization, we employed unsupervised network expansion to evaluate interaction pathways related to the function of splenic organization. After enrichment with 20 unknown connecting transcripts, a network associated with the regulation of spleen disorganization was constructed. While Cyclin dependent kinase inhibitor 2 A (*CDKN2A*) was found to be upregulated at 150 dpi (**Figure 5A**), *CXCL13*, *CCR5*, *CCL19*, *CCR6*, *CCR7* and *LTA* were downregulated, suggesting an inhibitory role played by *CDKN2A*. Interestingly, in our unsupervised analysis of canonical pathways, the consistent upregulation of *CDKN2A* indicated its involvement in the down-modulation of cell cycle regulation (**Figure 5B**).

## Discussion

The present work employed an experimental model of *L. infantum* infection in hamsters to evaluate sequential changes in spleen architecture during the course of VL. Hamsters are known to develop severe chronic disease, with similar clinicopathological changes that parallel those observed in human and canine VL. Here we show that these changes take place concurrently with the differential expression of regulatory transcripts that may interfere with leukocyte recruitment and could provoke cell cycle arrest. Our results suggest that *DLK1* and *CDKN2A* could both play central roles in the spleen disorganization observed in severe forms of VL. The data presented herein together with that previously published in the literature expands the observations to a chronic phase of VL, from 30 dpi to 150 dpi and enlighten about the possible pathways leading ultimately to spleen disorganization (5, 13, 18–20, 27, 36–38).

### Infection by *L. infantum* Induced Morphological Changes Leading to Spleen Disorganization in Hamsters

Several experimental animal models of infection have been used to study changes brought about by VL. In mice and hamsters, Veress et al. (11) observed the hyperplasia of lymphoid follicles during the progression of disease (11). In BALB/c mice, substantial tissue remodeling and changes in cell populations were found following infection by *L. infantum* or *L. donovani* (22, 27, 36, 38–40). However, the marked changes of the WP observed by other authors at early stages of infection did not progress to WP disruption at later stages of infection in mice (22, 27, 36, 38–40). In contrast, hyperplasia followed by atrophy and white pulp disruption was observed at later timepoints in the present experimental model involving hamsters. Veress et al. (22) observed complete WP disruption at around 120 days of infection, accompanied by extensive RP replacement by amyloid deposition (22). Herein, intense WP disruption was observed only at 150 dpi in the absence of amyloidosis. These differences may be associated with the *Leishmania* strain and inoculum size used in experimentation (38). Despite the sample size of seven animals per group, and the fact that hamster model is susceptible to severe forms of VL, not all the infected animals developed complete disruption of the WP during the time course of this study. In fact, in canine and human VL not all the individuals develop spleen disorganization, as reported by Veress et al. (22) and Santana et al. (6). Nevertheless, the *L. infantum* infection successfully induced splenic disorganization in 5 out of 7 animals at 120 dpi, and 3 out of 7 at 150 dpi. Further, 2 out of 7 animals had extensive disruption of the WP at 150 dpi. Our data indicate more exacerbated clinical VL manifestations at later stages of disease, as evidenced by weight loss, splenomegaly and a greater frequency and intensity of clinical signs.

### Transcript Expression Kinetics Uncovers *DLK1* Deregulation

The clinical and histological changes identified herein were found to be correlated with different transcriptomic signatures in accordance with the progression of infection. A normal resting spleen presents with balanced expression of genes involved in the maintenance of splenic compartments. Following infection, at 30 dpi, *DLK1* was found to be significantly differentially expressed concomitant with the histological detection of lymphoid hyperplasia. Previous studies have shown important events, such as cell population losses or mobilization from the usual environment between 14 and 30 days after experimental infection in mice (8, 12, 36). Along the course of the present experimental infection model, we observed a progressive decrease in *DLK1* expression, with WP disruption taking place by 150 dpi. Immunohistochemical staining for DLK1 corroborated the transcript expression and spleen disorganization. We chose an antibody-based validation for this finding, to reflect its biological relevance in the protein synthesis. As we show herein, cells expressing DLK1 decreases in density in the spleen over the course of infection. The *DLK1* gene is a member of a family of growth factors that regulate cell differentiation, and is highly expressed by CD34+ hematopoietic cells (41). *DLK1* has also been implicated in the differentiation of several cell types regulated by the Notch gene family (42). The absence of *DLK1* expression in *DLK1^-/-^* mice was shown to lead to decreased frequency of B cells in the lymphoid follicles, an increased presence of B cells in the MZ and greater IgG1 production (37). We therefore speculate that losses in follicular B cell populations, the accumulation of IgG-producing plasma cells in the RP and associated hypergammaglobulinemia previously reported by other authors may in fact be associated with alterations in *DLK1* expression as observed herein during the progression of infection (6, 13). Downregulation of *DLK1* was reported in mice infected with *L. donovani* at 21 and 42 days of infection (26). Although no correlation with spleen remodeling was investigated, it was shown that morphological changes in the spleen of mice with VL take place as early as 14 dpi. However, the spleen disorganization seems not to progress at later time points of the infection (8, 12, 27, 39, 40). In sum, correlations between changes in *DLK1* expression and the remodeling of splenic microenvironments seem to suggest the participation of this transcript in the regulation of B cell differentiation, likely leading to WP disruption and possibly even the accumulation of plasma cells in the RP.

### Splenic Transcriptomic Signatures Are Associated With Biological Functions of Leukocyte Trafficking and Granulocyte Recruitment

Transcriptomic processes involved in inflammatory response, leukocyte migration, granulocyte recruitment and lymphocyte chemotaxis were identified concomitantly with the detection of granulomas at 60 dpi. The establishment of infection at this stage could occur in response to the high expression of *IDO1* and *ARG1*. *IDO1* is induced by IFNγ and has been associated with the polarization of M2 macrophages in hamsters with VL (25). In *Leishmania* infection, *IDO1* also shows a function of suppressing innate immunity through regulatory stimulation of dendritic cells, and adaptive through the generation of regulatory T lymphocytes (43, 44). *ARG1* expression is induced in macrophages by *Leishmania*, which competes with iNOS for arginine and produces nutrients that favor parasite survival, such as urea, as well as decreasing the production of parasitotoxic nitric oxide (45). Altogether, these observations provide evidence of an inflammatory process at 60 dpi, which likely induced the T lymphocyte chemotaxis observed in the transcriptome at this stage.

T lymphocyte chemotaxis is notably high between 30 and 60 dpi, and granulocytes recruitment is increased, associated with differential expression of the genes presented in the **Figures 2**, **4**. Previous data from our group demonstrated that normal RP cellularity becomes replaced by high numbers of plasma cells in association with the elevated expression of *BAFF*, *APRIL* and *CXCL12* (3, 13). In fact, it is possible that granulocyte recruitment may be related to the presence of eosinophils in the splenic compartment, which could supply factors promoting the survival and maintenance of plasma cells at later times of infection (46).

A significant downmodulation in lymphocyte chemotaxis associated with the decreased expression of *IL12b* and *CCL21* was identified at 120 dpi, which correlated with the observation of moderate disorganization of splenic lymphoid tissue. Other authors previously reported a highly inflammatory environment in the spleen of hamsters infected by *L. donovani* (25). *IL12b* expression is associated with the polarization of a Th1 response against pathogens in the spleen, whereas *CCL21* expression is important for the retention of T lymphocytes in the periarteriolar zone of WP (29, 47). Alterations in the expression of these transcripts were correlated with decreasing tissue expression of CD3 in the PALS area, supporting a genetic regulation of splenic placement of T lymphocytes in the progression of the disease. Besides providing a validation of the alteration on splenic T lymphocytes found in the transcriptome at the cellular level, we further sought to provide insights on translation of the hamster model to the human disease. Using the nCounter method for deep-genome and multiplexed profiling, the same molecular signature was found in human VL. At 150 dpi we found profound disorganization of the spleen and decreased expression of transcripts involved in the organization of splenic microenvironments. The reduction of PALS in splenic tissue has been associated with decreased expression of *CCL19* and *CCR7*, the ligand of both *CCL19* and *CCL21* (40, 48). We also detected a progressive reduction in the expression of *LTα*, *CXCL13* and its receptor, *CXCR5*, along the course of infection. LTα is produced by stromal cells and is involved in WP formation during embryogenesis (29). In mice, the development of VL was marked by decreased number of stromal cells and impaired migration of dendritic cells within spleen compartments, both associated with low expression of *CCL19* and *CCL21* and attributed to a defective function of *CCR7* (48). In canine VL, despite the absence of alterations in *CCL19* and *CCL21* expression, splenic disorganization was found to be associated with a decreased frequency of CD3 T lymphocytes (18), T lymphocyte apoptosis (19) and T cell exhaustion (20). Moreover, a reduction in B cell density in lymphoid follicles was accompanied by a decreased *CXCL13* expression in dogs with VL and disorganized spleens (18).

### Transcriptomic Signature of Spleen Disorganization Is Associated With Cell Cycle Arrest

At the final stage of infection (150 dpi), the observed differentially expressed transcripts formed a network of gene regulation associated with spleen disorganization. When an unsupervised network expansion involving these transcripts was performed, a central link was identified with *CDKN2A*. Throughout the kinetics of gene expression shown herein, *CDKN2A* expression was observed to change from reduced (30 dpi) to an increased state at all timepoints after 60 dpi, coinciding with the development of an immunosuppressive environment in the spleen that resulted in poor immune response observed against *L. infantum*, and possibly other pathogens (49). Correspondingly, the expression of cytokines, chemokines and receptors associated with disorganization (*CXCL13*, *CCR5*, *CCL19*, *CCR6*, *CCR7* and *LTA*) predicted regulatory function by *CDKN2A* and was observed to significantly decline after 60 dpi. Immunohistochemistry staining corroborated the impairment of T lymphocyte chemotaxis between 120 and 150 dpi, leading to a reduction in PALS area, which may be potentially associated with the downregulation of *CDKN2A*-related genes as shown at 150 dpi. These changes are reflected on a tissue level, as evidenced by the fading of MZ, reduced PALS area and lymphoid follicle atrophy.

*CDKN2A* is known to transcribe information used in the production of proteins, such as p16 (INK4A) and p14 (ARF), both involved in cell cycle inhibition in the G1 phase (50). This transcripts also plays a role in cell senescence and is involved in immune inflammatory events leading to type I diabetes and atherosclerosis, in addition to being associated with inflammatory bowel disease (51, 52). Taken together, the associations reported herein suggest a regulatory role for *CDKN2A* with respect to cytokine-producing cells. *CDKN2A* was previously found upregulated in both hamster and mouse experimental models of *L. donovani* infection at earlier time points in comparison to this study (25, 26). Importantly, this transcript has been extensively investigated as a biomarker in some types of cancer (50), and may also constitute an early marker of severe outcomes in VL.

The present work explores transcriptional pathways involved in leukocyte chemotaxis, cell cycle arrest and possible alterations in B cell differentiation, in association with histological and clinical changes during the course of VL. The differential gene expression correlated with the phenotypical changes in the T lymphocytes in the spleen, and a similar pattern of the expression of transcripts involved in the spleen remodeling was found in severe cases of human VL. The transcriptome of human VL is, however, limited due to the lack of paired controls. The acquisition of healthy splenic tissue is difficult, since this organ is not usually submitted to biopsy. In some cases it is obtained from splenectomy due to trauma. We made use of the GTEx database (33), which is well established and used by many others. Differential expression of *DLK1* and *CDKN2A* emerging with potential regulatory roles in spleen disorganization is novel in the field and requires further investigations to confirm its relevance. Our results serve to affirm, on a transcriptomic and histological level, the central role played by the spleen in the establishment of infection and disease progression and stand in agreement with previous phenomenological observations associating a severe profile of VL with disorganization of the spleen.

## Data Availability Statement

The datasets presented in this study can be found in online repositories. The names of the repository/repositories and accession number(s) can be found below: https://www.ncbi.nlm.nih.gov/, PRJNA695421.

## Ethics Statement

The studies involving human participants were reviewed and approved by Instituto Gonçalo Moniz IGM-FIOCRUZ. The patients/participants provided their written informed consent to participate in this study. The animal study was reviewed and approved by Instituto Gonçalo Moniz.

## Author Contributions

CM, MH and WS designed the animal experiments. CM, MH, JF, BM, and RB performed the experiments. RK, FG, PR, and GF designed the transcriptomic study and RK, FG, and CM conducted the analysis. LF, CC, and WS obtained the human samples and conducted the histopathological analysis. CM and WS wrote the manuscript. All authors contributed to the article and approved the submitted version.

## Funding

The authors acknowledge the financial support provided by the Conselho Nacional de Desenvolvimento Científico e Tecnológico – CNPq (grant no. 400905/2013-2) and the research productivity scholarship provided to WS. We are also thankful for financial support from the Coordenação de Aperfeiçoamento de Pessoal de Nível Superior – CAPES (grant no. 2072_2013) and the Fundação de Amparo à Pesquisa do Estado da Bahia – FAPESB (no. PET0053_2013).

## Conflict of Interest

The authors declare that the research was conducted in the absence of any commercial or financial relationships that could be construed as a potential conflict of interest.

## Publisher’s Note

All claims expressed in this article are solely those of the authors and do not necessarily represent those of their affiliated organizations, or those of the publisher, the editors and the reviewers. Any product that may be evaluated in this article, or claim that may be made by its manufacturer, is not guaranteed or endorsed by the publisher.

## References

[1] Melby PC Chandrasekar B Zhao W Coe JE . The Hamster as a Model of Human Visceral Leishmaniasis: Progressive Disease and Impaired Generation of Nitric Oxide in the Face of a Prominent Th1-Like Cytokine Response. J Immunol (2001) 166:1912–20. doi: 10.4049/jimmunol.166.3.1912 11160239

[2] Moreira N das D Vitoriano-Souza J Roatt BM Vieira PM de A Ker HG Cardoso JM de O . Parasite Burden in Hamsters Infected With Two Different Strains of Leishmania (Leishmania) Infantum: “Leishman Donovan Units” Versus Real-Time PCR. PloS One (2012) 7:e47907. doi: 10.1371/journal.pone.0047907 23112869 PMC3480442

[3] Hermida M d’El-R de Melo CVB Lima IDS Oliveira GGdeS Dos-Santos WLC . Histological Disorganization of Spleen Compartments and Severe Visceral Leishmaniasis. Front Cell infection Microbiol (2018) 8:394. doi: 10.3389/fcimb.2018.00394 PMC6243053 30483481

[4] Lage DP Ludolf F Silveira PC MacHado AS Ramos FF Dias DS . Screening Diagnostic Candidates From Leishmania Infantum Proteins for Human Visceral Leishmaniasis Using an Immunoproteomics Approach. Parasitology (2019)146:1467–76. doi: 10.1017/S0031182019000714 31142384

[5] Lima IS Silva JS Almeida VA Leal FG Souza PAN Larangeira DF . Severe Clinical Presentation of Visceral Leishmaniasis in Naturally Infected Dogs With Disruption of the Splenic White Pulp. PloS One (2014) 9:e87742. doi: 10.1371/journal.pone.0087742 24498367 PMC3911999

[6] Santana CC Vassallo J De Freitas LAR Oliveira GGS Pontes-De-Carvalho LC Dos-Santos WLC . Inflammation and Structural Changes of Splenic Lymphoid Tissue in Visceral Leishmaniasis: A Study on Naturally Infected Dogs. Parasite Immunol (2008) 30:515–24. doi: 10.1111/j.1365-3024.2008.01051.x PMC2592477 18665902

[7] Cavalcanti AS Ribeiro-Alves M De OR Pereira L Mestre GL Ferreira ABR . Parasite Load Induces Progressive Spleen Architecture Breakage and Impairs Cytokine mRNA Expression in Leishmania Infantum-Naturally Infected Dogs. PloS One (2015) 10:e0123009. doi: 10.1371/journal.pone.0123009 25875101 PMC4395300

[8] Engwerda CR Ato M Kaye PM . Macrophages, Pathology and Parasite Persistence in Experimental Visceral Leishmaniasis. Trends Parasitology (2004) 20:524–30. doi: 10.1016/j.pt.2004.08.009 15471704

[9] Varma N Naseem S . Hematologic Changes in Visceral Leishmaniasis/Kala Azar. Indian J Hematol Blood Transfusion (2010) 26:78–82. doi: 10.1007/s12288-010-0027-1 PMC3002089 21886387

[10] dos-Santos WLC Pagliari C Santos LG Almeida VA e Silva TLV Coutinho JdeJ . A Case of Conventional Treatment Failure in Visceral Leishmaniasis: Leukocyte Distribution and Cytokine Expression in Splenic Compartments. BMC Infect Dis (2014) 14:1–7. doi: 10.1186/1471-2334-14-491 25200768 PMC4175220

[11] Veress B Omer A Satir AA El Hassan AM . Morphology of the Spleen and Lymph Nodes in Fatal Visceral Leishmaniasis. Immunology (1977) 33:607. PMC1445510 590992

[12] Engwerda CR Ato M Cotterell SEJ Mynott TL Tschannerl A Gorak-Stolinska PMA . A Role for Tumor Necrosis Factor-α in Remodeling the Splenic Marginal Zone During Leishmania Donovani Infection. Am J Pathol (2002) 161:429–37. doi: 10.1016/S0002-9440(10)64199-5 PMC1850733 12163368

[13] Silva-O’Hare J De Oliveira IS Klevorn T Almeida VA Oliveira GGS Atta AM . Disruption of Splenic Lymphoid Tissue and Plasmacytosis in Canine Visceral Leishmaniasis: Changes in Homing and Survival of Plasma Cells. PloS One (2016) 11:e0156733. doi: 10.1371/journal.pone.0156733 27243459 PMC4887081

[14] Fox CH Cottler-Fox M . The Pathobiology of HIV Infection. Immunol Today (1992) 13:353–6. doi: 10.1016/0167-5699(92)90171-3 1361326

[15] Urban BC Hien TT Day NP Phu NH Roberts R Pongponratn E . Fatal Plasmodium Falciparum Malaria Causes Specific Patterns of Splenic Architectural Disorganization. Infect Immun (2005) 73:1986–94. doi: 10.1128/IAI.73.4.1986-1994.2005 PMC1087405 15784539

[16] Duarte-Neto AN Monteiro RAA da Silva LFF Malheiros DMAC de Oliveira EP Theodoro-Filho J . Pulmonary and Systemic Involvement in COVID-19 Patients Assessed With Ultrasound-Guided Minimally Invasive Autopsy. Histopathology (2020) 77:186–97. doi: 10.1111/his.14160 PMC7280721 32443177

[17] Kaneko N Kuo HH Boucau J Farmer JR Allard-Chamard H Mahajan VS . Loss of Bcl-6-Expressing T Follicular Helper Cells and Germinal Centers in COVID-19. Cell (2020) 183:143–57.e13. doi: 10.1016/j.cell.2020.08.025 32877699 PMC7437499

[18] Silva JS Andrade AC Santana CC Santos LQ de Oliveira CI Veras PST . Low CXCL13 Expression, Splenic Lymphoid Tissue Atrophy and Germinal Center Disruption in Severe Canine Visceral Leishmaniasis. PloS One (2012) 7:e29103. doi: 10.1371/journal.pone.0029103 22242159 PMC3252310

[19] de Lima VMF Fattori KR de Souza F Eugênio FR dos Santos PSP Rozza DB . Apoptosis in T Lymphocytes From Spleen Tissue and Peripheral Blood of L. (L.) Chagasi Naturally Infected Dogs. Vet Parasitol (2012) 184:147–53. doi: 10.1016/j.vetpar.2011.08.024 21899954

[20] de Souza TL da Silva AVA de OR Pereira L Figueiredo FB Mendes Junior AAV Menezes RC . Pro-Cellular Exhaustion Markers Are Associated With Splenic Microarchitecture Disorganization and Parasite Load in Dogs With Visceral Leishmaniasis. Sci Rep (2019) 9:1–14. doi: 10.1038/s41598-019-49344-1 31506501 PMC6736856

[21] Sacks DL Melby PC . Animal Models for the Analysis of Immune Responses to Leishmaniasis. Curr Protoc Immunol (2015) 108:19.2.1–.24. doi: 10.1002/0471142735.im1902s108 25640990

[22] Veress B Abdalla RE El Hassan AM . Visceral Spreading Depletion of Thymus-Dependent Regions and Amyloidosis in Mice and Hamsters Infected Intradermally With Leishmania Isolated From Sudanese Cutaneous Leishmaniasis. Br J Exp Pathol (1983) 64(5):505–14. PMC2040808 6605763

[23] Medina-Colorado AA Osorio EY Saldarriaga OA Travi BL Kong F Spratt H . Splenic CD4+ T Cells in Progressive Visceral Leishmaniasis Show a Mixed Effector-Regulatory Phenotype and Impair Macrophage Effector Function Through Inhibitory Receptor Expression. PloS One (2017) 12:e0169496. doi: 10.1371/journal.pone.0169496 28103263 PMC5245871

[24] Perez LE Chandrasekar B Saldarriaga OA Zhao W Arteaga LT Travi BL . Reduced Nitric Oxide Synthase 2 (NOS2) Promoter Activity in the Syrian Hamster Renders the Animal Functionally Deficient in NOS2 Activity and Unable to Control an Intracellular Pathogen. J Immunol (2006) 176:5519–28. doi: 10.4049/jimmunol.176.9.5519 16622021

[25] Kong F Saldarriaga OA Spratt H Osorio EY Travi BL Luxon BA . Transcriptional Profiling in Experimental Visceral Leishmaniasis Reveals a Broad Splenic Inflammatory Environment That Conditions Macrophages Toward a Disease-Promoting Phenotype. PloS Pathog (2017) 13:e1006165. doi: 10.1371/journal.ppat.1006165 28141856 PMC5283737

[26] Ashwin H Seifert K Forrester S Brown N MacDonald S James S . Tissue and Host Species-Specific Transcriptional Changes in Models of Experimental Visceral Leishmaniasis. Wellcome Open Res (2018) 3:135. doi: 10.12688/wellcomeopenres.14867.1 30542664 PMC6248268

[27] de Melo CVB Hermida MDER Mesquita BR Fontes JLM Koning JJ da S Solcà M . Phenotypical Characterization of Spleen Remodeling in Murine Experimental Visceral Leishmaniasis. Front Immunol (2020) 11:653. doi: 10.3389/fimmu.2020.00653 32351510 PMC7174685

[28] Cesta MF . Normal Structure, Function, and Histology of the Spleen. Toxicol Pathol (2006) 34:455–65. doi: 10.1080/01926230600867743 17067939

[29] Mebius RE Kraal G . Structure and Function of the Spleen. Nat Rev Immunol (2005) 5:606–16. doi: 10.1038/nri1669 16056254

[30] Ewels P Magnusson M Lundin S Käller M . MultiQC: Summarize Analysis Results for Multiple Tools and Samples in a Single Report. Bioinformatics (2016) 32:3047–8. doi: 10.1093/bioinformatics/btw354 PMC5039924 27312411

[31] Dobin A Davis CA Schlesinger F Drenkow J Zaleski C Jha S . STAR: Ultrafast Universal RNA-Seq Aligner. Bioinformatics (2013) 29:15–21. doi: 10.1093/bioinformatics/bts635 23104886 PMC3530905

[32] Robinson MD McCarthy DJ Smyth GK . Edger: A Bioconductor Package for Differential Expression Analysis of Digital Gene Expression Data. Bioinformatics (2009) 26:139–40. doi: 10.1093/bioinformatics/btp616 PMC2796818 19910308

[33] Lonsdale J Thomas J Salvatore M Phillips R Lo E Shad S . The Genotype-Tissue Expression (GTEx) Project. Nat Genet (2013) 45:580–5. doi: 10.1038/ng.2653. PMC4010069 23715323

[34] Durinck S Spellman PT Birney E Huber W . Mapping Identifiers for the Integration of Genomic Datasets With the R/ Bioconductor Package biomaRt. Nat Protoc (2009) 4:1184–91. doi: 10.1038/nprot.2009.97 PMC3159387 19617889

[35] Anders S Pyl PT Huber W . HTSeq-A Python Framework to Work With High-Throughput Sequencing Data. Bioinformatics (2015) 31:166–9. doi: 10.1101/002824 PMC4287950 25260700

[36] Smelt SC Engwerda CR McCrossen M Kaye PM . Destruction of Follicular Dendritic Cells During Chronic Visceral Leishmaniasis. J Immunol (1997) 158(8):3813–21. 9103448

[37] Raghunandan R Ruiz-Hidalgo M Jia Y Ettinger R Rudikoff E Riggins P . Dlk1 Influences Differentiation and Function of B Lymphocytes. Stem Cells Dev (2008) 17:495–507. doi: 10.1089/scd.2007.0102 18513163 PMC3189718

[38] Carrión J Nieto A Iborra S Iniesta V Soto M Folgueira C . Immunohistological Features of Visceral Leishmaniasis in BALB/c Mice. Parasite Immunol (2006) 28(5):173–83. doi: 10.1111/j.1365-3024.2006.00817.x 16629702

[39] Yurdakul P Dalton J Beattie L Brown N Erguven S Maroof A . Compartment-Specific Remodeling of Splenic Micro-Architecture During Experimental Visceral Leishmaniasis. Am J Pathol (2011) 179(1):23–9. doi: 10.1016/j.ajpath.2011.03.009 PMC3123882 21703391

[40] Ato M Maroof A Zubairi S Nakano H Kakiuchi T Kaye PM . Loss of Dendritic Cell Migration and Impaired Resistance to Leishmania Donovani Infection in Mice Deficient in CCL19 and CCL21. J Immunol (2006) 176(9):5486–93. doi: 10.4049/jimmunol.176.9.5486 PMC3602896 16622017

[41] Sakajiri S O’Kelly J Yin D Miller CW Hofmann WK Oshimi K . Dlk1 in Normal and Abnormal Hematopoiesis. Leukemia (2005) 19:1404–10. doi: 10.1038/sj.leu.2403832 15959531

[42] Falix FA Aronson DC Lamers WH Gaemers IC . Possible Roles of DLK1 in the Notch Pathway During Development and Disease. Biochim Biophys Acta - Mol Basis Dis (2012) 1822:988–95. doi: 10.1016/j.bbadis.2012.02.003 22353464

[43] Donovan MJ Tripathi V Favila MA Geraci NS Lange MC Ballhorn W . Indoleamine 2,3-Dioxygenase (IDO) Induced by Leishmania Infection of Human Dendritic Cells. Parasite Immunol (2012) 34:464–72. doi: 10.1111/j.1365-3024.2012.01380.x PMC3572781 22803643

[44] Makala LHC Baban B Lemos H El-Awady AR Chandler PR Hou DY . Leishmania Major Attenuates Host Immunity by Stimulating Local Indoleamine 2,3-Dioxygenase Expression. J Infect Dis (2011) 203:715–25. doi: 10.1093/infdis/jiq095 PMC3072725 21282196

[45] Gaur U Roberts SC Dalvi RP Corraliza I Ullman B Wilson ME . An Effect of Parasite-Encoded Arginase on the Outcome of Murine Cutaneous Leishmaniasis. J Immunol (2007) 179:8446–53. doi: 10.4049/jimmunol.179.12.8446 18056391

[46] Kita H . Eosinophils: Multifaceted Biological Properties and Roles in Health and Disease. Immunol Rev (2011) 242:161–77. doi: 10.1111/j.1600-065X.2011.01026.x PMC3139217 21682744

[47] Reeme AE Miller HE Robinson RT . IL12B Expression Is Sustained by a Heterogenous Population of Myeloid Lineages During Tuberculosis. Tuberculosis (2013) 93:343–56. doi: 10.1016/j.tube.2013.02.011 PMC4413898 23491716

[48] Ato M Stäger S Engwerda CR Kaye PM . Defective CCR7 Expression on Dendritic Cells Contributes to the Development of Visceral Leishmaniasis. Nat Immunol (2002) 3:1185–91. doi: 10.1038/ni861 12436111

[49] Costa CHN Werneck GL Costa DL Holanda TA Aguiar GB Carvalho AS . Is Severe Visceral Leishmaniasis a Systemic Inflammatory Response Syndrome? - A Case Control Study. Rev Soc Bras Med Trop (2010) 43:386–92. doi: 10.1590/S0037-86822010000400010 20802936

[50] Cánepa ET Scassa ME Ceruti JM Marazita MC Carcagno AL Sirkin PF . INK4 Proteins, a Family of Mammalian CDK Inhibitors With Novel Biological Functions. IUBMB Life (2007) 59:419–26. doi: 10.1080/15216540701488358 17654117

[51] Lee HS Lee SB Kim BM Hong M Jung S Hong J . Association of CDKN2A/CDKN2B With Inflammatory Bowel Disease in Koreans. J Gastroenterol Hepatol (2018) 33:887–93. doi: 10.1111/jgh.14031 29063720

[52] Martínez-Hervás S Sánchez-García V Herrero-Cervera A Vinué Á Real JT Ascaso JF . Type 1 Diabetic Mellitus Patients With Increased Atherosclerosis Risk Display Decreased CDKN2A/2B/2BAS Gene Expression in Leukocytes. J Transl Med (2019) 17:1–12. doi: 10.1186/s12967-019-1977-1 31299986 PMC6626385

