# Supplementary material for: Splenic Transcriptional Responses in Severe Visceral Leishmaniasis: Impaired Leukocyte Chemotaxis and Cell Cycle Arrest
Source: Front Immunol. 2021 Nov 5;12:716314. doi: 10.3389/fimmu.2021.716314 (PMC8602831; doi:10.3389/fimmu.2021.716314)
Supplement: Supplementary file 1 [file DataSheet_1.docx]

# **Supplementary Material**


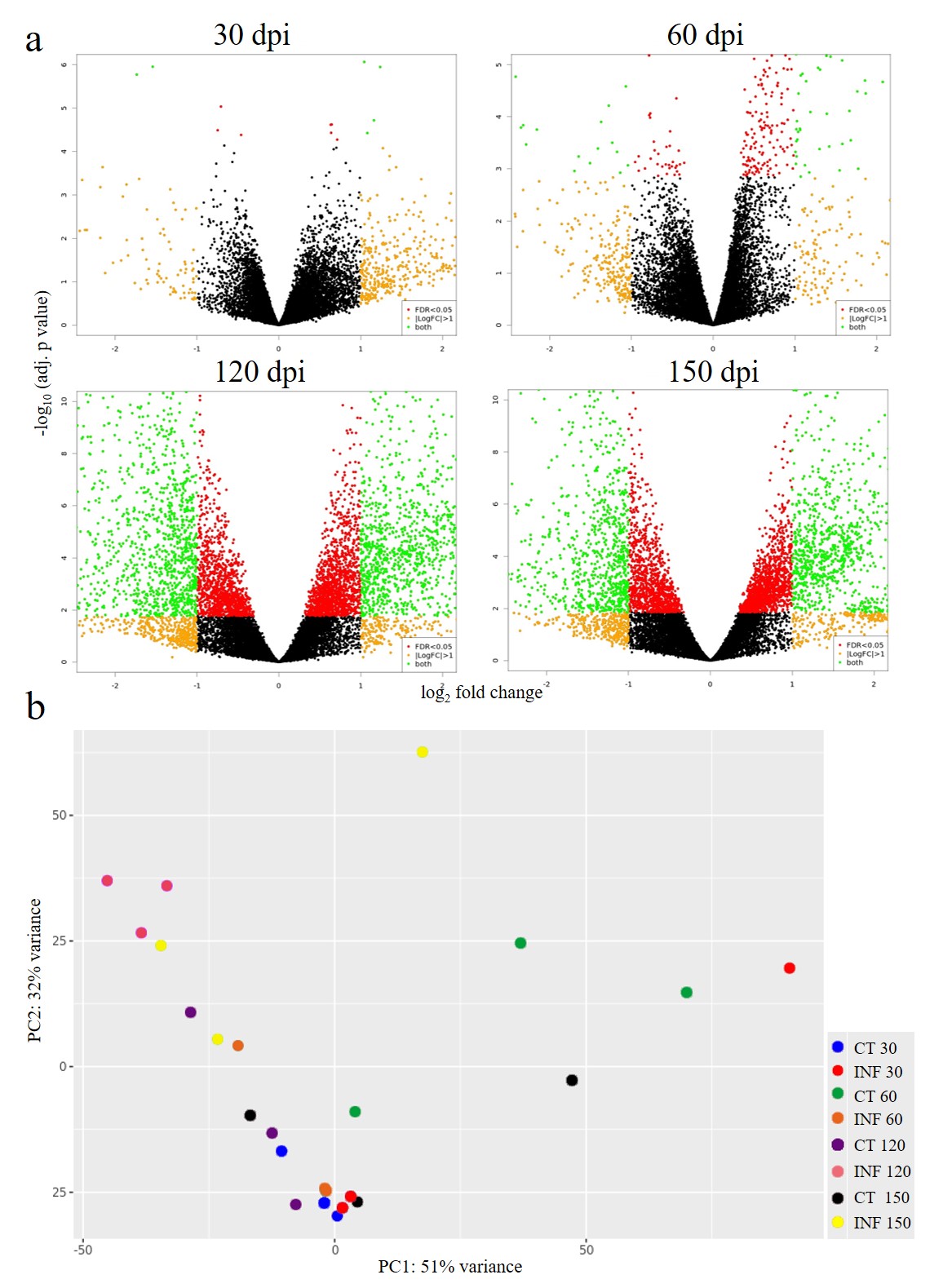


**Figure S1. Transcriptomic analysis of splenic response in *Leishmania infantum* infection. (A)** Differential transcripts (relative to uninfected hamsters) represented in volcano plots as log2 FC against log10 adjusted p values. Red dots represent FDR<0.05, yellow dots represent logFC>1 and green dots represent both FDR<0.05 and logFC>1. **(B)** Principal component analysis of spleen transcriptomic profile of control (CT) and infected groups (INF). 30 dpi= blue (control), red (infected); 60 dpi= green (control), orange (infected); 120 dpi= purple (control), pink (infected); 150 dpi= control (black), yellow (infected). Data are represented as PC1 (51% variance) vs. PC2 (32% variance).

**Table S1. Functional analysis of canonical pathways from** **splenic tissue in *Leishmania infantum*-infected and uninfected hamsters.**

| Canonical Pathways | 30dpi | 60dpi | 120dpi | 150dpi |
| --- | --- | --- | --- | --- |
| Cyclins and Cell Cycle Regulation | N/A | N/A | 2 | 1,897 |
| Estrogen-mediated S-phase Entry | N/A | N/A | 2 | 1,633 |
| SPINK1 Pancreatic Cancer Pathway | N/A | 3 | N/A | N/A |
| Heme Biosynthesis II | N/A | N/A | N/A | 2,236 |
| VDR/RXR Activation | N/A | N/A | N/A | 2,236 |
| GP6 Signaling Pathway | N/A | N/A | N/A | -2 |

Note: Numbers represent log2FC of infected animals in comparison to control groups. N/A= not applicable.

**Table S2. Enrichment analysis of diseases and functions from** **splenic tissue in *Leishmania infantum*-infected and uninfected hamsters.**

| Diseases and Bio Functions | 30dpi | 60dpi | 120dpi | 150dpi |
| --- | --- | --- | --- | --- |
| Chemotaxis of T lymphocytes | N/A | 2,927 | 1,678 | 1,854 |
| Quantity of Ca2+ | N/A | 1,665 | 1,985 | 2,581 |
| Recruitment of granulocytes | N/A | 2,412 | 2,359 | 0,975 |
| Quantity of metal ion | N/A | N/A | 2,301 | 2,918 |
| Delayed hypersensitive reaction | N/A | 2 | 1,433 | 1,576 |
| Movement of CD4+ T-lymphocytes | N/A | 2,2 | 1,052 | 1,553 |
| Cell movement of T lymphocytes | N/A | 2,525 | 1,037 | 1,119 |
| Migration of dendritic cells | N/A | 2,195 | 0,933 | 1,36 |
| Migration of CD4+ T-lymphocytes | N/A | 1,982 | N/A | 2,183 |
| Infection of mammalia | N/A | -1,359 | -2,645 | N/A |
| Fibrosis | N/A | 2,051 | -0,876 | -0,892 |
| Recruitment of mononuclear leukocytes | N/A | 2,207 | 0,902 | 0,666 |
| Urination disorder | N/A | -0,323 | -2,26 | -1,157 |
| Proliferation of bone marrow cells | N/A | N/A | 1,633 | 2,059 |
| Quantity of metal | N/A | N/A | 2,034 | 1,603 |
| Chemotaxis | N/A | 2,25 | N/A | 1,197 |
| Growth of tumor | N/A | 1,163 | 2,174 | N/A |
| Hemorrhagic disease | N/A | N/A | -1,069 | -2,153 |
| Recruitment of leukocytes | N/A | 2,416 | N/A | 0,738 |
| Inflammatory response | N/A | 2,75 | 0,078 | 0,257 |
| Cell movement of leukocytes | N/A | 2,035 | N/A | 1,05 |
| Recruitment of myeloid cells | N/A | 2,043 | N/A | 1,012 |
| Seizure disorder | N/A | N/A | N/A | 3,054 |
| Seizures | N/A | N/A | N/A | 3,046 |
| Melanoma | N/A | N/A | 2 | 0,456 |
| Congenital malformation of genitourinary system | N/A | N/A | N/A | -2,449 |
| Ingestion by rodents | N/A | N/A | N/A | -2,444 |
| Emotional behavior | N/A | N/A | N/A | -2,386 |
| Ingestion by mice | N/A | N/A | N/A | -2,275 |

Note: Numbers represent log2FC of infected animals in comparison to control groups. N/A= not applicable.

**Table S3. Human spleen transcriptomics of patients with visceral leishmaniasis and healthy donors.**

| Gene name | VL1 | VL2 | VL3 | HealthyDonors |
| --- | --- | --- | --- | --- |
| SPP1 | 2,878739555 | 2,372602233 | 4,841545357 | 1,556 |
| IL12B | 6,553735943 | 5,896164189 | 6,120093845 | 8,2 |
| IL21 | 9,92555444 | 10,6295843 | 9,489346241 | 7,954 |
| IFNG | 4,668166597 | 4,434355676 | 5,059176543 | 2,05 |
| CXCL9 | 5,646154456 | 6,618685943 | 5,943674439 | -2,474 |
| CXCL10 | 4,976425032 | 6,264453824 | 6,551545454 | -4,644 |
| CXCL11 | 4,943870309 | 5,556883978 | 5,692965333 | 0,6135 |
| CCL5 | 2,363142328 | 0,202143564 | 0,98121101 | 3,812 |
| CCL24 | 0,008933125 | -1,760864059 | -0,466409116 | 3,385 |

Note: Numbers represent log2FC of human patients (VL1, VL2 and VL3) in comparison to healthy donors.
